# Chemical Composition and Antimicrobial Properties of *Piper ovatum* Vahl

**DOI:** 10.3390/molecules14031171

**Published:** 2009-03-16

**Authors:** Daniel Rodrigues Silva, Eliana Harue Endo, Benedito Prado Dias Filho, Celso Vataru Nakamura, Terezinha Inez Estivaleti Svidzinski, Amanda de Souza, Maria Claudia M. Young, Tânia Ueda-Nakamura, Diógenes Aparício Garcia Cortez

**Affiliations:** 1 Programa de Pós-graduação em Ciências Farmacêuticas, Universidade Estadual de Maringá, Maringá, Paraná, Brazil; 2 Departamento de Análises Clínicas, Universidade Estadual de Maringá, Maringá, Paraná, Brazil; 3 Instituto de Botânica de São Paulo, São Paulo, SP, Brazil; 4 Departamento de Farmácia e Farmacologia, Universidade Estadual de Maringá, Maringá, Paraná, Brazil

**Keywords:** *Piper ovatum* Vahl, Chemical composition, Essential oil, Antimicrobial activity.

## Abstract

The chemical composition of the essential oil obtained from the leaves of *Piper ovatum* Vahl by hydrodistillation was analyzed by GC–MS. The main constituents found were *δ*-amorphene (16.5 %), *cis*-muurola-4(14),5-diene (14.29 %) and γ-muurolene (13.26%). The crude extracts and isolated compounds were screened for their antimicrobial activity. Hydroalcoholic extracts of different parts of *Piper ovatum* Vahl, essential oil and amides isolated from leaves were tested against Gram-positive and Gram-negative bacteria and *Candida* species. All extracts and amides were active against *Bacillus subtilis* and *Candida tropicalis,* including clinical strains. Essential oil was active against *C. tropicalis*. These amides showed an inhibitory effect on the adherence of *C. tropicalis* ATCC 28707 on cover glasses at 10 µg/mL, but did not show morphological alterations at the tested concentrations. Amides were identified as piperovatine and piperlonguminine, and showed MIC values of 15.6 and 31.2 µg/mL to *B. subtilis* and 3.9 µg/mL to *C. tropicalis,* and low toxic effects to Vero cells and macrophages.

## 1. Introduction

Medicinal plants are an important source of natural compounds with biological properties, including antimicrobial effects. Due to the occurrence of resistance to antimicrobials and the incidence of infectious diseases, there is a need to search for new antimicrobial compounds that may inhibit microorganisms by different mechanisms than those in current use [1,2]. 

*Piper ovatum* Vahl (Piperaceae), an herbaceous plant occurring throughout Brazil, is popularly known as “joão burandi” or “anesthetic.” It is used in traditional medicine for the treatment of inflammations and as an analgesic [3]. The genus *Piper* includes about 700 species, which are widely distributed in tropical and subtropical regions. Because they contain some substances with biological activity, several species of *Piper* have been studied, and the presence of amides, lignanes, neolignanes, flavonoids, phenolic, terpenes and steroid compounds has been reported [4,5]. 

An amide isolated from *Piper* species was piperine, which possesses analgesic and anti-inflammatory activity [6]. Antimicrobial properties have also been reported for other *Piper* species: *P. lanceafolium* showed antifungal activity against *Candida albicans*, and extracts of *P. regnellii* showed antibacterial and antifungal activity [7,8]. 

Piperovatine was isolated from the leaves of *P. alatabaccum* [9]. Piperlongumine, piperovatine, isopiperlonguminine, corcovadine and isocorcovadine were obtained from *Ottonia frutens.* Piperovatine is also present in other species, such as *Ottonia frutescens* roots and bark, *P. piscatorum* roots, and *P. longum* fruits [10,11]. Piperovatine and piperlonguminine from *P. ovatum* leaves showed excellent activity against amastigote and promastigote forms of *L. amazonensis*, and the mixture of these two amides showed an anti-inflammatory effect in an ear model in mice compared with the indomethacin control [12,13].

Antifungal activity of essential oils extracted from *Piper aduncum, Piper arboreum* and *Piper tuberculatum* leaves, bark and fruits against *Cladosporium sphaerospermum* and *Cladosporium cladosporioides* has been reported [14]. *P. multiplinervium* showed an antimicrobial effect against *Helicobacter pylori, Staphylococcus aureus, Escherichia coli, Klebsiella pneumoniae, Mycobacterium smegmatis, Pseudomonas aeruginosa* and *Candida albicans* [15]. 35 compounds have been identified by CG and CG-MS analysis of *Ambrosia trifida* oil, which showed antimicrobial activity against bacteria and fungi [16].

*Candida* species are harmLess saprophyte yeasts, components of the normal human biota in the gastrointestinal tract and oral and vaginal mucosae. These yeasts can cause superficial infections such as thrush and vaginitis; however, if the immune defenses of the host become compromised, they can cause severe systemic infections. Although *C. albicans* is the most common fungal pathogen, infections associated with non-*albicans* species have been increasing. *Candida tropicalis* is the third most common species isolated [17,18].

Studies of plants as a source of therapeutic agents should be emphasized. In the present study, we identified active substances and essential oil compounds obtained from *Piper ovatum* Vahl, and investigated their antimicrobial activity *in vitro.*

## 2. Results and Discussion

**Figure 1:**
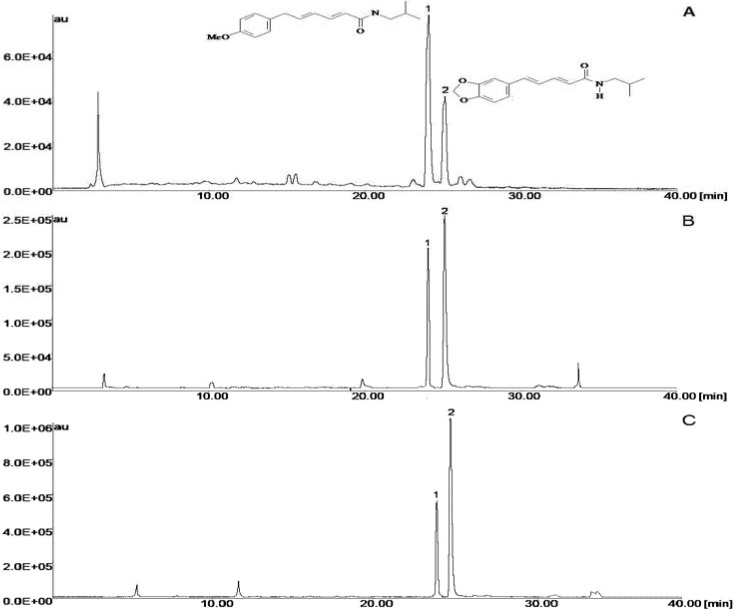
Chromatograms of hydroalcoholic extracts of stems (A), leaves (B) and root (C) *Piper ovatum*; where the piperovatine (1), piperlonguminine (2). Chromatographic conditions: Metasil ODS column; mobile phase: acetonitrile:water 0 % of acetonitrile for 60 % in 30 minute and acetonitrile:water 60:40 (v/v) in isocratic for 10 minute, with 1% acetic acid; ﬂow rate: 1.0mL/min; room temperature; detection: 280 nm.

Active fractions obtained from leaves of *Piper ovatum* were identified as piperovatine (**1**) and piperlonguminine (**2**) by analyses of their ^1^H- and ^13^C-NMR data of and comparison with data from the literature [13,19]. Figure 1 shows the chromatogram of the crude extracts. Peak 1, with a retention time of 23.50 min, was identified as piperovatine. Peak 2, with a retention time of 24.46 min, was piperlonguminine. For component identification, the essential oil was submitted to Gas Chromatography and Mass Spectrometry (GC/MS) analysis, and the substances identified are listed in Table 1. The components isolated in the highest quantities were *δ*-amorphene (16.5 %), *cis*-muurola-4(14),5-diene (14.29 %) and γ-muurolene (13.26%). The oil obtained from *P. ovatum* was found to be composed of approximately equal amounts of monoterpene hydrocarbons (6.82%), oxygenated monoterpenes (2.27%), oxygenated sesquiterpene (27.27%), sesquiterpene hydrocarbons (52.27%), ketones (4.54%) and unidentified compounds (6.82%).

**Table 1:** Main components of the essential oil from the aerial parts of *P. ovatum* Vahl.

Peak No.	Components	Área(%)	IK	IK*	Identification Method
1	*α*-Pinene	0.27	930	939	RT GC MS
2	*β*-Pinene	0.38	1020	1024	RT GC MS
3	Sylvestrene	0.45	1024	1030	RT GC MS
4	Terpinyl acetate	0.21	1334	1349	RT GC MS
5	Silphiperfol-4,7(14)-diene	2.34	1348	1360	RT GC MS
6	*α*-Copaene	4.55	1376	1376	RT GC MS
7	*α*-Dihydroionone	0.14	1384	1412	RT GC MS
8	Longifolene	0.24	1390	1407	RT GC MS
9	*β*-Elemene	0.46	1392	1390	RT GC MS
10	(E)-Caryophyllene	8.49	1419	1419	RT GC MS
11	*β*-Gurjunene	1.76	1428	1433	RT GC MS
12	(E)- *α*-Ionone	0.19	1433	1430	RT GC MS
13	Aromadendrene	0.22	1436	1441	RT GC MS
14	*cis*-Calamenene	1.64	1448	1529	RT GC MS
15	*α*-Humulene	0.41	1450	1460	RT GC MS
16	Allo-Aromadendrene	0.33	1459	1460	RT GC MS
17	NI	1.46	1470		
18	γ- Muurolene	16.50	1476	1479	RT GC MS
19	*cis*-Muurola-4(14),5-diene	14.29	1478	1466	RT GC MS
20	γ-Gurjunene	0.39	1481	1477	RT GC MS
21	*trans*-Muurola-4(14),5-diene	1.15	1485	1493	RT GC MS
22	*δ*- Cadinene	4.89	1489	1523	RT GC MS
23	*α*-Muurolene	3.04	1494	1500	RT GC MS
24	*γ*-Cadinene	6.01	1508	1513	RT GC MS
25	*δ*-Amorphene	13.26	1518	1511	RT GC MS
26	*trans*-Cadina-1,4-diene	1.40	1525	1534	RT GC MS
27	*α*-Cadinene	0.49	1530	1538	RT GC MS
28	*α*-Calacorene	0.28	1535	1545	RT GC MS
29	Selina-3,7(11)-diene	0.37	1548	1546	RT GC MS
30	*trans*-Dauca-4(11),7-diene	0.26	1559	1557	RT GC MS
31	N.I.	0.38	1575		
32	*β*-Copaen-4*α*-ol	0.72	1579	1590	RT GC MS
33	Guaiol	0.20	1582	1600	RT GC MS
34	1,10-di- *epi*-Cubenol	0.33	1593	1619	RT GC MS
35	*γ*-Eudesmol	0.36	1608	1632	RT GC MS
36	*cis*-Cadin-4-en-7-ol	1.73	1620	1636	RT GC MS
37	N.I.	0.32	1624		
38	*epi*- *α*-Muurolol	2.77	1635	1642	RT GC MS
39	*α*-Muurolol	1.03	1639	1646	RT GC MS
40	*α*-Eudesmol	0.38	1644	1653	RT GC MS
41	*α*-Cadinol	2.03	1647	1654	RT GC MS
42	Bulnesol	0.24	1663	1671	RT GC MS
43	10-nor-Calamenen-10-one	0.71	1677	1702	RT GC MS
44	7,14-anhydro-Amorpha-4,9-diene	0.77	1738	1756	RT GC MS

T= Retention time; MS = mass spectra; GC=Gas Chromatography; KI- was calculated from the GC-MS chromatograms, Ki*-calculated using Adams data [20].

**Figure 2:**
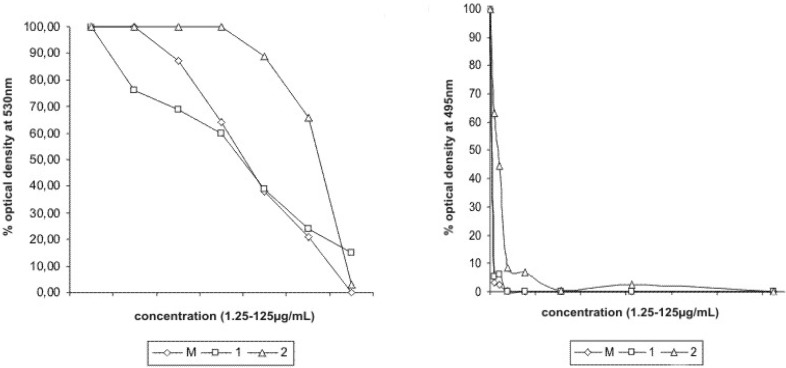
Growth inhibition of *Bacillus subtilis* ATCC 6623 and *C. tropicalis* ATCC 28707, by reduction on optical density at 530 nm and 495 nm, respectively.

**Figure 3:**
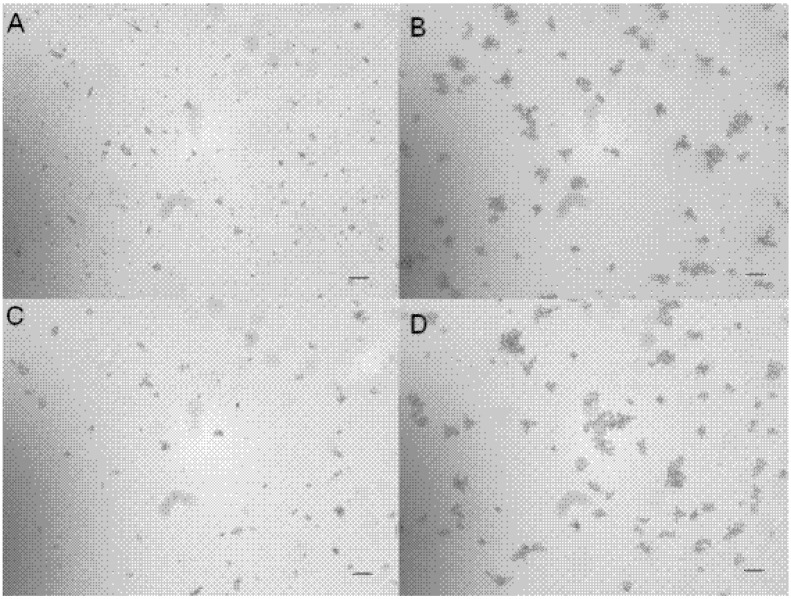
Adherence inhibition assay. A and C) *C. tropicalis* treated with 10 µg/mL of pipeovatine and piperlonguminine, respectively. B and D) *C. tropicalis* without treatment (control). Bars = 50µm.

Extracts from leaves, bark and roots of *Piper ovatum* were active against *B. subtilis* (250, 500 and 250 µg/mL, respectively) and *C. tropicalis* ATCC 28707 (500, 250 and 62.5 µg/mL, respectively) (Table 2). The isolated substances piperovatine and piperlonguminine showed good activity, with MIC values of 15.6 and 31.2 µg/mL, respectively, towards *B. subtilis* and of 3.9 µg/mL (both) towards *C. tropicalis* ATCC 28707. Piperlonguminine exhibited more activity then piperovatine against urine clinical isolates of *C. tropicalis*, with MIC 31.25 µg/mL (Table 2). Essential oil extracted from *P. ovatum* leaves showed an effect against *C. tropicalis* ATCC 28707 and *C. tropicalis* from urine clinical isolates (22.6±3.1 and 18.7 ±2.1 mm respectively, Table 3). Reduction of optical density at 530 nm and 495 nm indicated growth inhibition of *B. subtilis* and *C. tropicalis* at the tested concentrations from 0 to 125 µg/mL of piperovatine and piperlonguminine (Figure 2). Both piperovatine and piperlonguminine showed an effect on the adherence of *C. tropicalis* on cover glasses. When compared to untreated control yeasts, a decrease in the intensity of adhesion occurred in yeast treated with 10 µg/mL of the isolate (Figure 3).

**Table 2:** Minimal inhibitory concentration of *Piper ovatum* Vahl extracts and amides (µg/mL) and antibiotics used as a positive control.

Microrganisms	Origin	root	bark	leaves	1	2	Flu	Tetra
*E. coli*	ATCC 25922	>1000	>1000	>1000	>1000	>1000		1
*P. aeruginosa*	ATCC 27853	>1000	>1000	>1000	>1000	>1000		12.5
*E. cloacae*	ATCC 13047	>1000	>1000	>1000	>1000	>1000		12.8
*B. subtilis*	ATCC 6623	250	500	250	15.6	31.2		2.68
*S. aureus*	ATCC 25923	>1000	>1000	>1000	>1000	>1000		0.95
*S. epidermidis*	ATCC *12228*	>1000	>1000	>1000	>1000	>1000		1.9
*C. tropicalis*	ATCC 28707	62.5	250	500	3.9	3.9	1.9	
*C. albicans*	ATCC 10231	>1000	>1000	>1000	>1000	>1000	7.8	
*C. parapsilosis*	ATCC 22019	>1000	>1000	>1000	>1000	>1000	1.9	
*C. glabrata*	Urine	>1000	>1000	>1000	>1000	>1000	31.2	
*C. krusei*	Urine	>1000	>1000	>1000	>1000	>1000	62.5	
*C. tropicalis*	catheter tip				>1000	>1000	3.9	
*C. tropicalis*	Urine				250	250	3.9	
*C. tropicalis*	orotracheal tube				>1000	>1000	3.9	
*C. tropicalis*	Urine				500	500	3.9	
*C. tropicalis*	Urine				250	125	3.9	
*C. tropicalis*	Urine				125	62.5	3.9	
*C. tropicalis*	Urine				125	31.25	3.9	
*C. tropicalis*	Urine				250	250	3.9	

**1** (piperovatine); **2** (piperlonguminine); oil (essential oil); Flu (fluconazole) and Tetra (tetracycline)

**Table 3:** Antifungical activity (inhibition zone expressed in mm) investigated *Piper ovatum* Vahl essential oils and antibiotic used as a positive control.

Microrganisms	Origin	*Pure essential oil*	Nystatin
*C. tropicalis*	ATCC 28707	22.6±3.1	32 ± 2.7
*C. tropicalis*	catheter tip	14.3±1.1	27 ± 0.8
*C. tropicalis*	Urine	18.7 ±2.1	30 ± 0.3
*C. tropicalis*	orotracheal tube	13.8 ±0.8	23 ± 0.7
*C. tropicalis*	Urine	16.0 ±1.0	25 ± 1.3
*C. tropicalis*	Urine	11.9 ±1.1	35 ± 0.42
*C. tropicalis*	Urine	10.0±1.0	34 ±1.2
*C. tropicalis*	Urine	12.8 ±1.7	32 ± 2.1
*C. tropicalis*	Urine	14.0 ±0.7	26 ±0.7

The values represent the average of three determinations ± standard deviations

The effect on the morphology of *C. tropicalis* treated with amides extracted from *P. ovatum* was investigated by scanning electron microscopy. The MIC values for piperovatine were 15.2 and 3.9 µg/mL for *B. subtilis* and *C. tropicalis* respectively, and the values for piperlonguminine were 31.2 and 3.9 µg/mL, indicating a selective toxicity to these microorganisms. Many studies have been conducted on antimicrobial activity, including screenings of plants in search of antimicrobial properties [8]. Here we describe the isolation of active agents from *Piper ovatum* Vahl (Piperaceae) with antimicrobial effects.

In the analysis of the essential oil of *P. aduncum*, a total of 46 components were identified. The major component was identified as dill apiole or 4,5-dimethoxy-6-(2-propenyl)-1,3-benzodioxole (43.3 %), together with other minor components such as β-caryophyllene (8.3 %), piperitione (6.7 %) and α-humulene (5.1 %). Essential oil derived from *P. gibbilimbum* is dominated by the gibbilimbols A-D (74.2 %), with the remaining constituents being the terpenes camphene (13.6 %) and α-pinene (6.5 %) [20]. Data from the present study showed that the hydroalcoholic extract and the amides piperovatine and piperlonguminine from *P. ovatum* Vahl have good antimicrobial activity against *B. subtilis* and *C. tropicalis*. Essential oil was inhibitory to *C. tropicalis*. There is also an effect on adherence of *C. tropicalis* on glass, and low toxic effects to cells.

## 3. Conclusions

This report describes the isolation of the amides piperovatine and piperlonguminine, and the analysis of the chemical composition of essential oil obtained from *P. ovatum* leaves. Of the 41 compounds identified, those present in the largest quantities were *δ*-amorphene (16.5 %), *cis*-muurola-4(14),5-diene (14.29 %) and γ-muurolene (13.26%). Antimicrobial activity was observed against *B. subtilis,* and *C. tropicalis*, including clinical strains*.* Although there was a selective toxicity to fungal and bacterial cells, showing that this plant oil, extracts and pure compounds may have commercial potential as an antiseptic agent, further studies are necessary to elucidate the mechanism of antimicrobial action. 

## 4. Experimental

### 4.1. Plant material

Leaves of *Piper ovatum* Vahl were collected in December 2006 in Monte Formoso, state of Minas Gerais, Brazil, and were identified by Dr. Elsie Franklin Guimarães. A voucher specimen was deposited in the herbarium of the Department of Botany, University of Maringá (HUM 10.621).

### 4.2. Plant extraction and purification

Leaves were dried at room temperature and powdered (100 g). The extract was prepared by exhaustive maceration in ethanol-water (9:1 v/v) at room temperature, filtration, concentration under vacuum at 40°C to obtain a hydroalcoholic extract, and then lyophilization, which yielded 25 g of extract. The hydroalcoholic extract (14 g) was chromatographed in a vacuum silica-gel column, and eluted with gradients of hexane, dichloromethane-ethyl acetate (1:1 v/v), ethyl acetate and methanol, affording F1 (2.09 g), F2 (3.93 g), F3 (2.61 g) and F4 (3.70 g). The dichloromethane-ethyl acetate fraction F2 (3.93 g) was rechromatographed on a silica gel 60 (70-230 mesh) column chromatograph eluted with gradients of hexane, hexane/CH_2_Cl_2_ (98:2, 95:5, 90:10, 80:20 and 50:50 v/v), CH_2_Cl_2_, CH_2_Cl_2_/EtOAc (98:2, 95:5, 90:10, 80:20 and 50:50 v/v), EtOAc and MeOH, affording 108 fractions. Subfractions 23-38 (258.2 mg) were identified as mixtures of piperovatine and piperlonguminine. These subfractions were rechromatographed on a Sephadex LH 20, and the column chromatograph was eluted with ethyl acetate, obtaining 55 fractions. Fractions 13-30 (15 mg) and 39-48 (35 mg) were isolated and identified as piperovatine (**1**) and piperlonguminine (**2**) respectively, by analyses of ^1^H- and ^13^C –NMR data and comparison with data from the literature [13,19]. The NMR spectra were obtained on Bruker DRX-400 (8.4 T) and Varian Gemini 300 (7.05 T) spectrometers, using the deuterated solvent (CDCl_3_) TMS as the internal standard and a constant temperature of 298 K. Low-resolution electrospray data were acquired in the negative ion mode, using a Micromass Quattro-LC instrument. Silica gel 60 (70-230 and 230-400 mesh); TLC: silica gel plates F_254_ (0.25 mm thickness) were used for chromatographic separations.

### 4.3. Leaves distillation

*Piper ovatum* leaves (100 g) were hydrodistilled in a Clevenger-type apparatus for 3 h. The oil layers obtained were dried over anhydrous Na_2_SO_4_. The yields (2.3 % w/w) were averaged over three experiments, and calculated on the basis of the dry weight of the material. For GC studies, 1 mg of oil dissolved in 1.5 mL of hexane and 1 µL of solution was injected into the GC-MS spectrometer.

### 4.4. GC/MS analysis

For component identification, the essential oils were submitted to Gas Chromatography and Mass Spectrometry (GC/MS) analysis, performed using an Agilent GC (6890 Series) – quadrupole MS system (5973), equipped with a fused silica capillary column (30 m x 0.25 mm i.d. x 0.25 µm film, coated with DB-5), EI operating at 70 eV. Injector and detector temperatures were set at 250^o^C. The oven temperature program was 40°C for 1 min and 40- 240 °C at 3 °C/min, and helium was employed as the carrier gas (1 mL/min). The compound was identified by comparing retention indices [Kóvats Index (KI), determined relative to the retention times of a series of *n*-alkanes] [21] and mass spectra with literature data [22].

### 4.5. HPLC analysis

The HPLC analyses were carried out using a GILSON apparatus equipped with a quaternary pump (Pump 321), automatic injector valve (234) with 20 µL loop, degasifier (865), CTO-10Avp oven and a UV/visible detector model 152, controlled by a BOWTER computer program. In the chromatographic analysis, we used a reverse-phase column Metasil ODS, 5 µm, 150.0 x 4.6 mm, kept in an oven set at ambient temperature. HPLC conditions were as follows: solvent A, acetonitrile, and solvent B, 1.0 % acetic acid. A gradient elution used was 0–30 min, 0-60% A; 30–40 min, 60% A. Flow rate was 1.0 mL/min, and detection was at 280 nm. All the samples were prepared in triplicate. The reagents used to prepare the mobile phase were acetonitrile (HPLC grade from OmniSolv EM Science, Gibbstown, NJ), ultrapure water (Milli-Q system, Millipore, Bedford, USA), acetic acid (analytical grade, Merck, Darmstadt, Germany), and methanol (HPLC grade from OmniSolv EM Science, Gibbstown, NJ). The stock solutions of extracts of the leaves, stems and roots from *P. ovatum* were prepared in methanol at a concentration of 1,000 µg/mL. The solutions were filtered through a 0.45 µm membrane filter (Millipore, São Paulo, Brazil). 

### 4.6. Strains and growth conditions

The test microorganisms used included *Escherichia coli* ATCC 25922, *Pseudomonas aeruginosa* ATCC 27853, *Enterobacter cloaceae* ATCC 13047, *Bacillus subtilis* ATCC 6623, *Staphylococcus aureus* ATCC 25923, *Staphylococcus epidermidis* ATCC 12228, *Candida albicans* ATCC 10231, *C. tropicalis* ATCC 28707, *C. parapsilosis* ATCC 22019, and two clinical isolates of *C. glabrata* and *C. tropicalis* and one of *C*. *krusei*. Bacteria were maintained on Mueller Hinton Agar and subcultured in Mueller Hinton Broth before each experiment. Yeasts were maintained at 4 °C on Sabouraud Dextrose Agar plates and subcultured at 37 °C in Sabouraud Dextrose Broth before each experiment, to ensure viability and purity.

### 4.7. Microdilution MIC determination

The minimal inhibitory concentrations of the extract and oil for the strains were determined according to the M27-A2 and M7-A7 broth microdilution reference procedure of the NCCLS [23]. RPMI 1640 medium with L-glutamine without bicarbonate buffered with 0.165 M MOPS (morpholine propanesulfonic acid) was used for yeasts, and Mueller-Hinton broth for bacteria. Serial two-fold dilutions of the extracts and isolated substances were done in a microdilution plate (96 wells) containing 100 µL of sterile medium. Next, the inoculum was added to each well. The microplates were incubated at 37 °C for 48 h for yeasts and 24 h for bacteria. The MIC was defined as the lowest concentration that resulted in inhibition of visual growth. Minimal microbicidal concentrations were determined by subculturing 10 µL of the culture from each negative well and from the positive control, measured as described. 

### 4.8. Adherence inhibition assay

*C. tropicalis* (10^6^ CFU/mL) suspension, untreated (control) and treated with decimal dilutions of piperovatine and piperlonguminine (1,000 to 0.1 µg/mL) were aliquoted (500 µL) onto a 24-well plate containing round cover glasses. The plate was incubated at 37 °C for 1 h. The cover glasses were washed with sterile phosphate buffer saline (PBS) and observed with an inverted microscope.

### 4.9. Scanning electron microscopy

Yeasts treated with piperovatine and piperlonguminine (3.9 µg/mL) were fixed with 2.5% glutaraldehyde in 0.1 M cacodylate buffer, pH 7.2. Small drops of the fixed cells were placed on a specimen support with poly-L-lysine for 1 hour at room temperature. Subsequently, the samples were dehydrated in graded ethanol, critical-point dried in CO_2_, coated with gold, and examined on a Shimadzu SS-550 scanning electron microscope. Yeasts without treatment were also prepared.

### 4.10. Cytotoxicity assay

To investigate the cytotoxic effects of extract and isolated substances, confluent Vero and macrophage J774G8 cell monolayers grown in 96-well cell culture plates were incubated with different concentrations of extract, piperovatine and piperlonguminine for 48 h at 37 °C and 5% CO_2_. At the time, cultures fixed with 10% trichloroacetic acid for 1 h at 4 °C were stained for 30 min with 0.4% Sulforhodamine B (SRB) in 1% acetic acid and subsequently washed with distilled water. Bound SRB was solubilized with 150 µL 10 mM Tris-base solution. Absorbance was read in an ELISA plate reader at 530 nm. The cytotoxicity was expressed as a percentage of the optical density compared to the control.

### 4.11. Disc diffusion method

*In vitro* antifungal activity of the *P. ovatum* essential oil was determined by the agar disk diffusion method according to Rubio *et al.* [24]. Briefly, a suspension of each tested microorganism (2.0 mL of 10^5^ cells per mL) was carefully mixed in a tube with Mueller Hinton Agar (MHA, 18 mL), and then poured on Petri plates. Sterile filter-paper discs (Whatman No. 1, 6.0 mm in diameter) were impregnated with 15 µL of the oil and placed on the inoculated plates. Control disks containing 15 µL of the physiological saline and nystatin (100 U.I. or 20 µg/disc, Cecon, São Paulo, Brazil) were used. These plates were allowed to dry at room temperature for 2 h, and were incubated at 25ºC for 48 h. The diameters of the inhibition zones were measured in millimeters, and their means were calculated. All the tests were performed in triplicate, and the strains were tested, as listed in Table 3.
